# Nucleic acid-sensing toll-like receptors: Important players in Sjögren’s syndrome

**DOI:** 10.3389/fimmu.2022.980400

**Published:** 2022-10-31

**Authors:** Lena Alexopoulou

**Affiliations:** Aix Marseille Univ, CNRS UMR7280, INSERM U1104 Centre d’immunologie de Marseille-Luminy (CIML), Marseille, France

**Keywords:** Sjögren’s syndrome, TLR7, TLR8, TLR9, COVID-19, systemic lupus erythematosus, autoimmunity, nucleic acid-sensing TLRs

## Abstract

Sjögren’s syndrome (SS) is a chronic systemic autoimmune disease that affects the salivary and lacrimal glands, as well as other organ systems like the lungs, kidneys and nervous system. SS can occur alone or in combination with another autoimmune disease, such as systemic lupus erythematosus (SLE) or rheumatoid arthritis. The etiology of SS is unknown but recent studies have revealed the implication of the activation of innate immune receptors, including Toll-like receptors (TLRs), mainly through the detection of endogenous nucleic acids, in the pathogenesis of systemic autoimmune diseases. Studies on SS mouse models suggest that TLRs and especially TLR7 that detects single-stranded RNA of microbial or endogenous origin can drive the development of SS and findings in SS patients corroborate those in mouse models. In this review, we will give an overview of the function and signaling of nucleic acid-sensing TLRs, the interplay of TLR7 with TLR8 and TLR9 in the context of autoimmunity, summarize the evidence for the critical role of TLR7 in the pathogenesis of SS and present a possible connection between SARS-CoV-2 and SS.

## Introduction

Sjögren’s syndrome (SS) is a chronic systemic autoimmune disease characterized by lymphocytic infiltration of exocrine glands, along with salivary and lacrimal hypofunction, resulting in dryness of the mouth and eyes (1). Fatigue is also a common symptom in SS patients, and while SS is progressing, various organs and systems can be affected including lungs, joints, kidneys, liver, musculoskeletal and nervous system (2). The impaired function of the salivary glands in SS is most probably multifactorial, whereas interactions between the immune system and the epithelium contribute to disease initiation and the different stages of disease development in patients with SS (3, 4). The prevalence rates of primary SS vary considerably (0.09% - 2.7%) depending on the ethnicity and gender of the population studied as well as the classification criteria that have been revised many times (5). The disease has a strong female propensity, with a female to male ratio 9:1 and a peak incidence at the age of 50 years. The disease may be classified as primary SS when is encountered alone, or as secondary or associated SS when is associated with another autoimmune disorder, such as systemic lupus erythematosus (SLE), rheumatoid arthritis or systemic sclerosis (6). The heterogeneity in the clinical manifestation and pathophysiology of the diseases often leads to a delay in diagnosis. The etiology of SS is multifactorial with an interaction between environmental, genetic, epigenetic and hormonal factors.

SS and autoimmune diseases in general are the outcome of a dysregulated innate and adaptive immune system. Cells of the innate immune system and especially conventional dendritic cells (cDCs) and plasmacytoid dendritic cells (pDCs) establish a proinflammatory environment characterized by type I interferon (IFN) signature that enables propagation of the disease by further activating T and B cell subsets to produce autoantibodies that cooperate in glandular destruction (7, 8). Both systemic and localized autoimmune responses within the salivary gland are responsible for salivary gland dysfunction in SS. The systemic autoimmunity is highlighted by the detection of antinuclear antibodies (ANA), rheumatoid factor (RF), anti-Ro60/SSA, anti-Ro52/SSA, and anti-La/SSB autoantibodies, which are characteristic of primary SS and can be present years before salivary manifestations and/or diagnosis of primary SS disease (9, 10). The localized immune response of SS is manifested by ectopic lymphoid structures in the target glands consisting of infiltrating mononuclear cells, such as T and B lymphocytes, DCs and macrophages. The distribution of these infiltrating cells at the SS-lesions varies according to lesion severity, correlates with disease manifestations and in 30% of the SS patients the lymphoid structures develop in organized germinal center-like formations, establishing a structure for local production of antibodies (1, 11, 12). There is currently no known cure for SS, thus, understanding the underlying molecules and pathways that mediate SS is crucial in order to develop targeted treatments.

Although little is known about the mechanisms that support SS etiology, growing evidence points towards an important role of innate immune detection of nucleic acids by TLRs as well as cytosolic RNA and DNA sensors in the development of autoimmune diseases (13). These sensors survey different subcellular compartments and include the endosomal TLRs (in humans TLR3, TLR7, TLR8 and TLR9), the cytosolic RNA sensors retinoic acid-inducible gene I (RIG-I)-like receptors (RLRs) (RIG-I and MDA5) and the universal cytosolic DNA sensor cyclic GMP-AMP synthetase (cGAS) (13). Upon activation all these receptors mediate the transcriptional induction of type I IFNs and other genes that collectively initiate an antiviral host response. Moreover, the same receptors can be triggered upon detection of host-derived nucleic acids, which can lead to sterile inflammation and autoimmunity if their activation is uncontrolled (14).

The possible implication of RLRs and the cGAS-STING pathway in SS have been addressed in recent reviews (15, 16). Thus, here we will focus on recent data pointing to a pivotal role of the endosomal nucleic acid-sensing TLRs and especially TLR7 in SS pathogenesis. We will briefly introduce nucleic acid TLRs and present data for the interplay of TLR7 with TLR8 and TLR9 and its importance in autoimmunity. Then, we will highlight studies on nucleic acid TLR implication in SS mouse models, as well as in patients with primary SS. We will also present a possible connection between COVID-19 and SS through TLR7, and precent recent therapies that target the nucleic acid-sensing TLRs that might prove to be a novel strategy for preventing and or treating SS disease.

## Toll-like receptors

Toll-like receptors (TLRs) constitute a family of innate immune receptors that play a central role in the induction of innate and adaptive immunity. The name Toll (great in German) originates from a Drosophila mutant that was isolated in 1980 in search for genes implicated in fly embryo development (17). By now, thirteen TLRs have been reported in mammals and their role regarding ligand recognition and immune response has been advanced by studying experimental mouse model and mainly TLR-deficient mice (18). Mammalian TLRs detect conserved microbial components shared by many microorganisms (also known as pathogen-associated molecular patterns, PAMPs), as well as, endogenous components from dying or injured cells (also known as danger-associated molecular patterns, DAMPs). They show heterogeneous expression across innate and adaptive immune cells, as well as non-immune cells, like fibroblasts, epithelial and endothelial cells. TLR activation leads to recruitment of cytosolic adaptor molecules and activation of signaling pathways that result in the production of inflammatory cytokines, IFNs, chemokines and a variety of inducible proteins that can induce inflammation, immune regulation, cell survival and proliferation (19–21). Out of the 13 mammalian TLRs, TLR1 to TLR9 are conserved in humans and mice and within them four recognize nucleic acids of which TLR3 detects double-stranded RNA (22), TLR7 and TLR8 (human) recognize single-stranded RNA with distinct sequence preferences (23–25) and TLR9 senses DNA containing unmethylated CpG motifs (26). These nucleic acid-sensing TLRs are engaged not only in antimicrobial host defense, but also in the development of sterile-inflammation and autoimmune diseases through the detection of host-derived nucleic acids (27).

In order to enable detection of microbial-RNA and -DNA, while restricting detection of self- RNA and -DNA, nucleic acid-sensing TLRs are localized mainly intracellularly and regulated tightly (28). Indeed, endosomal localization, endosomal acidification and endosomal TLR proteolytic processing are essential for effective nucleic acid-sensing TLR activation (29). The multi-pass transmembrane protein Unc-93 homolog B1 (UNC93B1) is vital in coordinating the trafficking of nucleic acid-sensing TLRs from the endoplasmic reticulum to endolysosomes, where they meet their respective ligands and become activated (28, 30, 31). Indeed, UNC93B1 deficiency in mice or in humans leads to unresponsiveness to nucleic acid-sensing TLR ligands and increased susceptibility to specific viruses (32, 33). Moreover, UNC93B1 is important in stabilizing UNC93B1-dependent TLR proteins and preventing their degradation (34). In addition to UNC93B1, other molecules have also been identified as regulators for the trafficking and compartmentalization of certain nucleic acid-sensing TLRs, including S100A9 for TLR3, adaptor protein complex 4 (AP-4) for TLR7 and AP-2 for TLR9 (28). In pDCs the localization of TLR7 and TLR9 is further controlled by AP-3 (35, 36), while TLR7, but not TLR9, trafficking also depends on the small GTPase Arl8b and its effector Plekhm2, for type I IFN production (37, 38). The final destination of each nucleic acid-sensing TLR also dictates their signaling capacity, since different types of endosomes have the ability to induce the production of proinflammatory cytokines or type I IFNs (39). Thus, the trafficking routes and the compartments where the nucleic acid-sensing TLRs reside are quite diverse and complex, can vary between different cell types and determine their signaling capacity.

**Figure 1:**
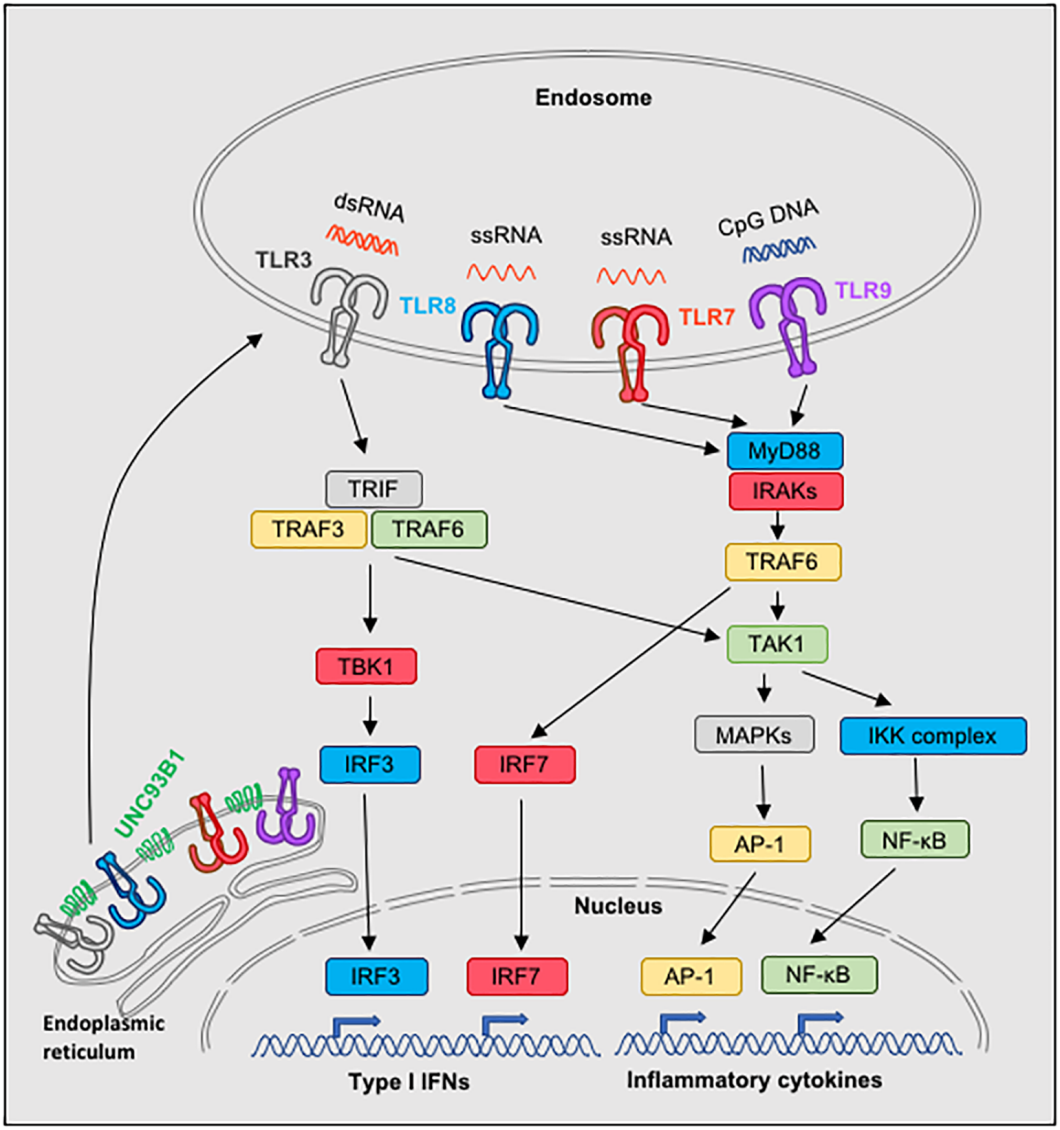
Signaling pathways of nucleic acid-sensing TLR3, TLR7, TLR8 and TLR9. Nucleic acid-sensing TLRs are translated in the endoplasmic reticulum and subsequently require the transmembrane protein UNC93B1 for exit and trafficking via the classical secretory pathways to endosomes. On top of UNC93B1, each TLR is associated with additional molecules and is transported to designated compartments by individual mechanisms, which can vary depending on the cell type (not illustrated). Once in endosomes and upon ligand processing and binding to the appropriate TLR, signaling pathways are activated: TLR7, TLR8 and TLR9 use the MyD88 pathway, whereas TRIF is used in the TLR3 signaling. Both MyD88 and TRIF mediate activation of NF-kB and subsequend induction of inflammatory cytokine genes. Moreover, MyD88 is responsible for TLR7-, TLR8- and TLR9-dependent activation of IRF7, and TRIF for TLR3-dependent activation of IRF3, which lead to the induction of type I IFNs.

TLRs are type I transmembrane proteins with leucine-rich repeats extracellular for ligand recognition, a transmembrane domain and an intracellular toll-interleukin 1 (IL-1) receptor (TIR) domain that is required for the activation of downstream signaling transduction pathways (40, 41). Five different signaling adaptor molecules can be recruited by distinct TLRs, which are MyD88 (myeloid differentiation primary-response protein 88), TRIF (TIR domain-containing adaptor protein inducing IFN-β), TIRAP (TIR-associated protein), also known as MAL, TRAM (TRIF-related adaptor molecule) and SARM (sterile α-and armadillo-motif-containing protein), and trigger distinct signaling pathways. MyD88 is used by all TLRs, except TLR3, while TRIF is used by TLR3 and TLR4 (19, 21). TRAM is recruited specifically to TLR4, but not TLR3, and serves as a link between TLR4 and TRIF, while SARM is a negative regulator of the MyD88- and TRIF-dependent TLR signaling (19, 21). From all the TLRs, only TLR4 is able to bind and signal through MyD88 and TRIF, which can lead to the production of proinflammatory cytokines and type I IFNs, although the two pathways utilize different intermediate molecules and have different kinetics. Thus, each TLR is using a specific combination of adaptor molecules and triggers a number of downstream molecules activating signaling cascades that lead to NF-κB, IRFs and MAPKs activation and induce the transcription of various immune response genes, including inflammatory cytokines, type I IFNs, chemokines and costimulatory molecules. The precise downstream signaling complexes for each TLR have been reviewed in detail previously (19, 21), and thus will not be presented here. A simplified view of the signaling pathways of TLR3, TLR7, TLR8 and TLR9 is presented in **Figure 1**.

## Interplay of TLR7 with TLR8 and TLR9 and its importance in autoimmunity

The primary role of the TLRs is to detect microbial components and rapidly signal innate and subsequently adaptive immune responses to fight infections and provide memory for future protection. Thus, when the idea of an intra-regulation between the TLRs was emerged it has been viewed with quite some skepticism. Nevertheless, by now it is undoubtedly documented that TLR7 plays a central role in SLE, where both TLR8 and TLR9 restrain TLR7 signaling and thus, can protect from autoimmunity. The importance of this phenomenon is presented below.

In SLE autoantibodies against nucleic acids are a hallmark of the disease. Thus, as soon as it was discovered that TLR7 and TLR9 detect viral and bacterial nucleic acids, various studies deployed to evaluate their role in autoimmunity, predominately by studying experimental mouse models. Primary studies using different lupus-prone mouse strains crossed to TLR7- or TLR9-deficient mice, revealed that depletion of TLR7 ameliorates SLE, while surprisingly ablation of TLR9 exacerbates the disease (42–45). In accordance, overexpression of TLR7 in a non-lupus prone background revealed that a 2-fold increase in TLR7 expression is sufficient for the development of spontaneous SLE in mice, whereas a modest increase of TLR7 expression promotes autoreactive lymphocytes with RNA specificities and proliferation of myeloid cells, while substantial increase of TLR7 expression leads to profound dendritic cell (DC) dysregulation and fatal acute inflammation (46). Moreover, deletion of TLR7 in lupus prone male mice bearing the Y chromosome-linked autoimmune accelerating (Yaa) locus that harbors 17 genes (including TLR7), demonstrated that TLR7 gene duplication was the cause for the development of lupus in these mice (46, 47). The mystery of why deletion of TLR9 in lupus prone mice leads to exacerbation of SLE was resolved by studying the correlation between TLR7 and TLR9. In lupus-prone TLR9-deficient mice the enhanced lupus disease is associated with functionally upregulated expression of TLR7 by B cells and pDCs, whereas the disease is suppressed upon TLR7 deletion (48, 49). Importantly, TLR9-deficiency in the non-lupus prone C57BL6 mouse background also leads to the development of a spontaneous lupus, a phenotype that has been overlooked since the lupus disease is quite milder than the one that develop lupus prone mouse models that carry various diseases elements (50). In addition, *in vitro* studies with HEK293 cells transfected with human TLRs in a pairwise combination showed that human TLR9, as well as human TLR8, can inhibit TLR7 signaling (51). Thus, these studies clearly demonstrated that TLR9 restrain TLR7 expression and signaling, and solved the mystery of why TLR9-deficiency in lupus prone mice leads to enhanced disease, despite the fact that results in reduction of anti-DNA antibodies.

Both in humans and mice the TLR7 and TLR8 genes reside on the X chromosome. In contrast to human TLR7, human TLR8 and murine TLR7 that detect ssRNA, murine TLR8 is unable to do so due to a five amino acid deletion in its extracellular part (24, 52, 53). This discrepancy between mouse and human TLR8 led to an initial belief that murine TLR8 was not functional. Nevertheless, our studies on TLR8-deficient mice revealed that TLR8 has an important function on restraining TLR7 and protecting from autoimmunity. Indeed, TLR8-deficient mice on the C57BL/6 background, which is not prone to lupus, develop SLE due to increased TLR7 expression and signaling by cDCs and pDCs (54). Moreover, TLR8 deletion in the Nba2.Yaa lupus prone mice accelerates SLE due to increased TLR7 responses (55). Not only the deletion, but also the overexpression of TLR8 affects TLR7 expression. Indeed, expression of human TLR8 in mice leads to the downregulation of endogenous mouse TLR7 levels (56). Importantly, the significance of TLR8 on restraining TLR7 has been recently outlined in humans. A case study revealed that a germline missense mutation (G572V) in the TLR8 gene in identical male twins, led to reduced TLR8 protein levels in monocytes and granulocytes and increased TLR7 signaling, which caused severe autoimmune hemolytic anemia and autoinflammation (57). Interestingly, inhibition of TLR7 signaling by hydroxychloroquine addition to the therapeutic regimen resulted in marked clinical improvement. Moreover, the patient’s mother, whereas half of her cells (due to random inactivation of the X chromosome) had also reduced levels of TLR8 and increased response to TLR7 ligands, had a history of polyarthritis and steroid-sensitive antiphospholipid syndrome (57). Thus, these results clearly demonstrate that TLR8 restrains TLR7 expression and signaling and protects from autoimmunity not only in mice, but also in humans.

**Figure 2:**
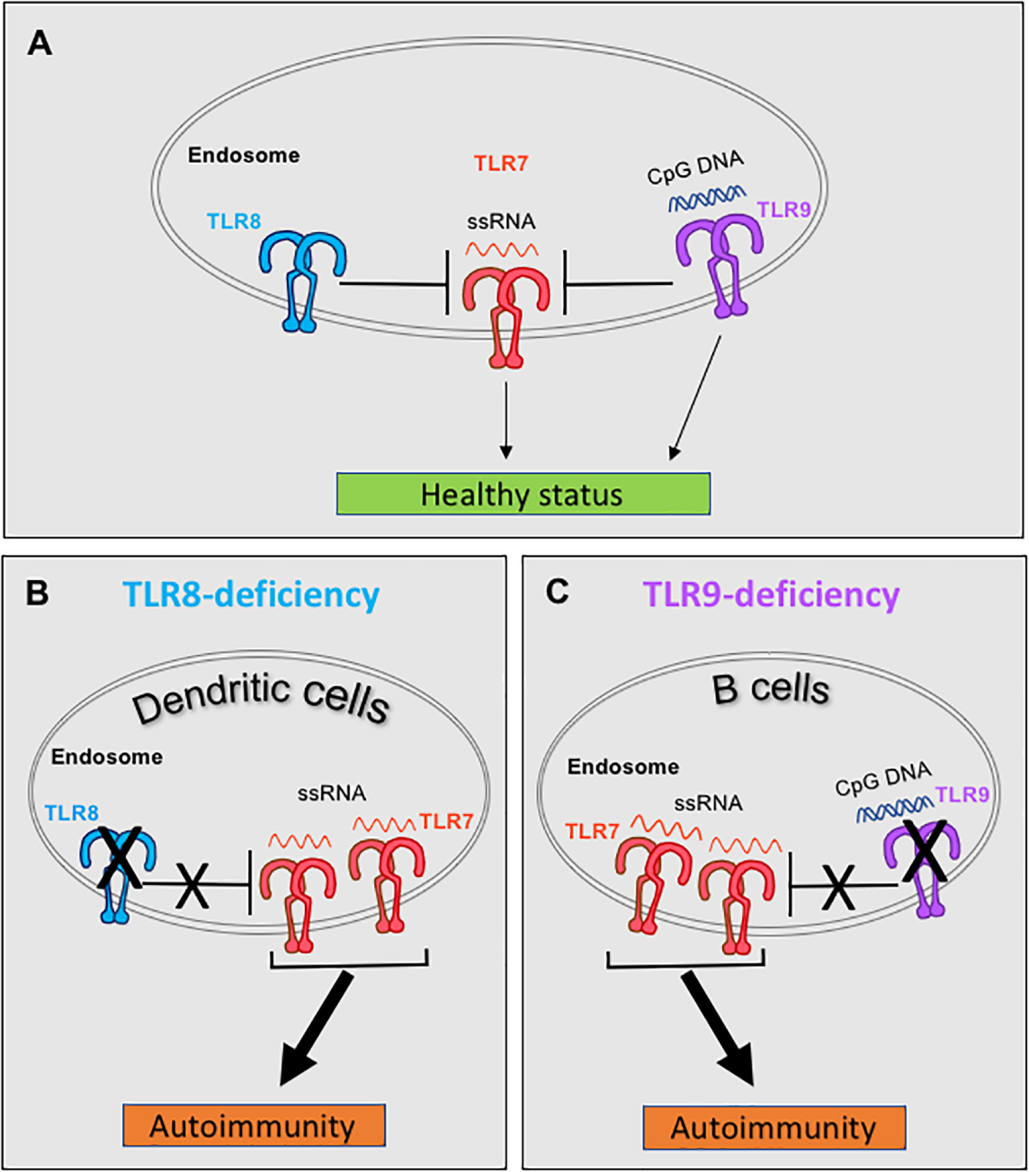
Murine TLR8 and TLR9 restrain TLR7 in dendritic cells (DCs) and B cells respectively, and protect from spontaneous autoimmunity. **(A)** Under normal conditions both TLR8 and TLR9 restrain TLR7 expression and signaling. **(B)** In C57BL/6 mice, deletion of TLR8 leads to increased expression of TLR7 in DCs, which leads to increased TLR7 signaling and development of SLE and Sjögren’s syndrome. **(C)** Deletion of TLR9 increases the number of TLR7 molecules in the endosomes (while the overall cellular TLR7 expression remains unchanged), which enables increased TLR7 signaling by B cells in TLR9-deficient C57BL/6 mice and the development of SLE. Discussion on similar control of TLR7 by TLR8 and TLR9 in humans are provided in the main text, in the section "Interplay of TLR7 with TLR8 and TLR9 and its importance in autoimmunity".

Therefore, both TLR8 and TLR9 are able to restrain TLR7 and their deletion leads to autoimmunity due to increased TLR7 responses. But what is the relation between TLR8 and TLR9 in controlling TLR7? This was addressed, by parallel evaluation of the SLE phenotype in single TLR8-, single TLR9- and double TLR8/9-deficient mice on the C57BL/6 background. Double TLR8/9-deficient mice have exacerbated SLE disease compared to single TLR8- or TLR9-deficient mice, unveiling that TLR8 and TLR9 have an additive effect on controlling TLR7 (50). Evaluation of TLR7 expression and responses to TLR7 ligand (R848) in various cell types, revealed that TLR8-deletion leads to increased expression and signaling of TLR7 in DCs (pDCs and cDCs), while TLR9-deletion drives an increase TLR7 signaling in B cells, without affecting overall B cell TLR7 expression levels (50, 54). In accordance to our studies, a recent report confirmed that in the MRL/lpr lupus-prone mice, TLR9 deficiency in B cells is sufficient to exacerbate lupus nephritis, whereas TLR9 deficiency in pDCs, cDCs, macrophages, monocytes and/or neutrophils has no effect in disease development (58). Thus, in mice TLR8 and TLR9 have an additive effect on controlling TLR7 in a cell type dependent manner, where TLR8 restrains TLR7 on DCs, while TLR9 contains TLR7 on B cells (**Figure 2**).

What are the mechanisms by which TLR8 and TLR9 restrain TLR7? TLR7 and TLR9 compete for UNC93B1-dependent trafficking, whereas TLR9 predominates since it has a higher affinity for UNC93B1 than TLR7 (59). Interestingly, a D34A mutation of UNC93B1 that affects the binding capacity of TLR9 to UNC93B1 does not influence the overall TLR7 or TLR9 expression, but leads to increased TLR7 trafficking from the endoplasmic reticulum to endolysosomes, where TLR7 becomes activated. Knock-in mice that carry the UNC93B1 D34A mutation develop TLR7-dependent systemic lethal inflammation (splenomegaly, thrombocytopenia and hepatitis with fibrosis) due of increased response to TLR7 ligands and signaling (60). Thus, UNC93B1 controls homeostatic TLR7 activation by balancing TLR9 to TLR7 trafficking, and we can hypothesize that a similar mechanism exists between TLR8 and TLR7. Overall, TLR8- or TLR9-deficiency decreases the competition with TLR7 for association with UNC93B1 and results to increased TLR7 trafficking and signaling that ultimately leads to autoimmunity.

## Lessons from mice on the implication of nucleic acid-sensing TLRs in SS

Despite the differences between humans and mice, established mouse models of SS displaying disease features similar to those of human SS and are valuable since they accelerate the elucidation of disease mechanisms (61). Findings from mouse studies regarding the possible implication of surface located TLRs, including TLR1, TLR2, TLR4, TLR6 and TLR5 in SS development have been reviewed previously (62, 63). Here, we will focus mainly on recent advancements regarding the contribution of nucleic acid-sensing TLRs in SS disease in SS mouse models.

## NOD mice: Type I diabetes and SS

The Non-obese diabetic (NOD) mouse has been extensively used as a mouse model for studying SS. The NOD mice develop type I diabetes, as well as SS-like manifestations that occur in a sex-specific manner, with males spontaneously developing lacrimal gland inflammation and females spontaneously developing salivary gland inflammation (sialadenitis) around the age of 10 weeks (64–66). Moreover, the development of autoimmune SS in NOD mice depends on commensal microbiota, whereas under germ-free conditions 13 weeks old female nondiabetic NOD mice show reduced sialadenitis compared with specific pathogen-free female NOD mice (67). Evaluation of the expression of all the murine TLRs (TLR1-TLR9 and TLR11-TLR13), as well as MyD88 in the submandibular glands (SMGs) of female NOD mice aged 4 up to 16 weeks, demonstrated that mRNA levels of TLR1, TLR2, TLR4, TLR9 and MyD88 were upregulated starting at the age of 8 weeks, while TLR3 levels were overexpressed only at the age of 16 weeks. Moreover, administration of chloroquine, which inhibits the activation of endosomal nucleic acid-sensing TLRs (68), in NOD mice starting at 4 weeks of age, abolished the elevated expression of TLR1, TLR2, TLR3, TLR4 and TLR9 and reduced sialadenitis in 16 weeks old mice (69). Thus, TLRs and MyD88 seem to be involved in the development and/or progression of the SS disease in NOD mice.

MyD88 is required for TLR signaling (except TLR3) and is critical for SS development. MyD88 deficiency in NOD mice, as well as in lupus-prone B6/lpr mice that also develop spontaneous autoimmune sialadenitis, leads to a clearly reduced SS pathology, including lymphocytic infiltration and production of proinflammatory cytokines (67, 70). Furthermore, microarray analysis of gene expression profiling of the SMGs of 10 weeks old female MyD88-deficient NOD versus NOD mice revealed that MyD88 deficiency mainly led to downregulation of genes that are involved in autoimmune diseases, like SLE and rheumatoid arthritis, as well as IFN-associated immunopathological processes (71).These data suggest that signaling through MyD88 is pivotal for SS development. However, it is important to note that MyD88 serves as a critical signaling adaptor downstream not only of TLRs, but also of IL-1R, IL-18R and IL-33R (19, 21). Hence, the observed phenotypes in MyD88-deficient mice may not be due only to the lack of TLR signaling, but may also reflect the absence IL-1R, IL-18R and/or IL-33R signaling.

Since TLR7 and TLR9 are critical in SLE development some studies focused on determining whether TLR7 or TLR9 activation could contribute to sialadenitis in NOD mice. To this end, administration of the oligonucleotide BL-7040, which activated TLR9 to induce an alternative NF-κB activation, in female NOD and TLR9-deficient mice led to amelioration of SS in NOD mice, including increased salivation and elevated anti-inflammatory responses in salivary glands, while TLR9-deficient mice were resistant to the salivation-promoting effect of BL-7040 (72). On the contrary, administration of the IFN-α-activating CpG-A oligonucleotide, which signals through TLR9, triggered a decline in salivary function. Hence, activation of TLR9 by BL-7040 could be beneficial in treating SS. In another study, evaluation of TLR9 and its downstream p38 MAPK signaling pathway in female NOD mice showed that starting from 5 weeks and up to 10 weeks of age, double positive TLR9 and p38 MAPK peripheral blood mononuclear cells (PBMCs) were increased in NOD mice and this increase was accompanied by reduced stimulated salivation and the presence of TLR9 or p38 MAPK positive infiltrating lymphocytes in the SMG of NOD mice (73). Thus, depending on the experimental settings and type of activation TLR9 signaling might have a beneficial or detrimental effect on the development and/or progression of sialadenitis and salivation in NOD mice.

The assessment of the contribution of TLR7 in the development of SS in the NOD mice, was done by deleting TLR7 in the NOD strain through CRISP/Cas9-mediated gene editing (74). Female TLR7-deficient NOD mice started developing diabetes at the age of 15 weeks, while male TLR7-deficient NOD mice did not develop diabetes (74). Evaluation of lacrimal and salivary gland inflammation in 10 weeks old mice, revealed that male TLR7-deficient NOD mice were protected from lacrimal inflammation, but surprisingly developed salivary gland inflammation compared to NOD mice (74). In contrast, female NOD mice developed focal sialadenitis regardless of the presence or absence of TLR7, while lacrimal gland inflammation was absent. In addition, RNA sequencing analysis on lacrimal glands derived from male NOD or TLR7-deficient NOD mice revealed that TLR7 signaling largely drove a type I IFN response. These data suggest different disease mechanisms in the development of SS and type 1 diabetes manifestations in male and female NOD mice, and depending on the tissue, TLR7 can promote or protect from disease manifestations in male mice, while it has no effect in female mice.

## NOD-relevant mouse substrains: models of primary SS

Depending on the source of the mice and the environment, the development of type I diabetes in NOD mice can vary between the two genders regarding penetrance and age of onset (75), and that has consequences also for the SS phenotype in NOD mice since salivary gland dysfunction is associated with hyperglycemia and systemic inflammation (76). Therefore, although NOD mice have been extensively used on studying SS, the interpretation of the results is not always straight forward due to the interconnection of the SS with the diabetes phenotype. Hence, pSS mouse models are useful for the discovery of the distinct mechanisms of the disease, and some of them have been derived from the NOD strain. One such pSS mouse model is the C57BL/6.NOD-Aec1Aec2 mice, which were generated by breeding two genetic intervals (associated with Idd3 and Idd5) from the NOD mice into non-autoimmune C57BL/6 mice (77). These mice fully manifest the SS-like phenotype of the NOD strain, including decreased salivary and lacrimal gland secretory flow, appearance of autoantibodies and glandular lymphocytic focal infiltrates, but without any signs of autoimmune diabetes (77). Microarray analysis on submandibular RNA isolated from female C57BL/6.NOD-Aec1Aec2 pre-diseased (8 weeks) mice revealed upregulation of TLR3 and TLR7, as well as their downstream signaling molecules IRF3, IRF5, IRF7 and TRAF6 that are associated type I IFN production (78). However, in diseased (12 weeks) mice the genes that were upregulated were associated with B cell activation, proliferation and differentiation (78). Hence, the data from this mouse model suggest that in the early stages of SS the signaling pathways of the nucleic acid-sensing TLR3 and TLR7 are activated.

Another mouse model of pSS that has been derived from the NOD strain is the NOD.B10 (NOD.B10Sn-H2b/J) mice. These mice were generated by replacing the NOD diabetogenic MHC locus, with that of the healthy C57BL/10S strain and the resultant congenic mice are protected from diabetes, but still develop SS (79). The NOD.B10 mice have a female disease predisposition and develop spontaneous SS by the age of 6 months, characterized by lymphocytic infiltrates in the salivary and lacrimal glands, reduced salivation and increased antinuclear antibodies (80, 81). Evaluation of SS in female NOD.B10 mice deficient for MyD88 demonstrated attenuated SS disease, that was characterized by a reduction of lymphocytic infiltrations in exocrine tissues, lungs and kidneys, normal salivation and diminished total and ANA specific autoantibody titers (82). Moreover, RNA sequencing analysis of splenocytes derived from clinical diseased 6 months old female NOD.B10 mice showed increased expression of TLR1, TLR6, TLR8, TLR12 and TLR13, as well as cytokines and chemokines that are associated to TLR signaling pathways (83). In the same study, further analysis of splenic and salivary tissues demonstrated that female NOD.B10 mice had increased TLR2 and TLR4 responses, which were attenuated in MyD88-deficient NOD.B10 mice. In addition, evaluation of autoantibodies against degraded extracellular matrix constituents were found to be diminished in MyD88-deficient NOD.B10 mice (84). Thus, MyD88 signaling contributes to the local and systemic inflammation of pSS in NOD.B10 mice.

## Secondary or associated SS in SLE mouse models

SLE and SS can occur together, whereas 14-17.8% of people living with SLE also develop SS (85). The reason for this coexistence is unknown, but probably there are common mechanisms that lead to SLE and SS based on some common features of the two diseases, including genetic and environmental risk factors, similar autoantibodies, clinical manifestations and hormones (85). Not only in humans, but also in mice SLE can coexist with SS, as it is the case for MRL/lpr mice (86). These mice have been extensively used to dissect the role of nucleic acid-sensing TLRs in lupus, however, only few studies have studied the role of TLRs in the development of associated SS in this mouse model.

MRL/lpr mice encompasses the key features of SS including lymphocytic infiltrates in the salivary and lacrimal glands, decreased salivation, anti-Ro/SSA and anti-La/SSB antibody production and female sex predisposition (86, 87). Evaluation of autoimmunity in female MyD88-deficient MRL/lpr (MyD88-/- MRL/lpr) mice at 24 weeks of age revealed reduced severity of salivary gland inflammation and serum titers of anti-dsDNA autoantibodies, as well decreased autoimmune nephritis, compared to MD88+/- MRL/lpr mice (88). These findings suggest that MyD88 signaling plays a crucial role in the development of sialadenitis in MRL/lpr mice. Furthermore, 12 weeks old TLR9-deficient MRL/lpr mice develop more accelerated end-organ disease in the salivary glands, characterized by the development of significant mononuclear infiltrate in the periacinar region of the salivary glands, higher levels of anti-DNA autoantibodies, and more sever lupus than MRL/lpr mice (44). As we mentioned before TLR9 restrains TLR7 and TLR9-deficiency leads to increased TLR7 signaling by B cells (50). Accordingly, in the MRL/lpr mice, TLR9-deficiency in B cells, but not in other major immune cell types, is sufficient to exacerbate lupus nephritis (58). Hence, we can hypothesize that the accelerated sialadenitis and autoimmune phenotype in the TLR9-deficient MRL/lpr mice is due to the increased TLR7 signaling by B cells, however, this aspect was not addressed in the study.

TLR8-deficient mice in the C57BL/6 background develop spontaneous SLE due to increased TLR7 expression and signaling by DCs (50, 54, 89). Interestingly, these mice also develop spontaneous SS (90). We found that the SS development in TLR8-deficient mice is manifested by sialadenitis, increased serum titers of anti-SSA, anti-SSB and anti-RNA autoantibodies, IgG immunocomplex deposition in the salivary glands and lung inflammation (90). This phenotype was observed in both genders, but it was more exacerbated in female than in male mice, which coincides with the fact that TLR7 expression is higher in female TLR8-deficient salivary glands and DCs than in male mice (90, 91). Moreover, increased cytokine and chemokine production (IL-6, TNF, LTα, CXCL13, CXCR5, BAFF), as well as ectopic lymphoid structures containing B cells, T cells, DCs, and high endothelial venules, were characteristic in the salivary glands of TLR8-deficient mice. Most importantly, deletion of TLR7 in TLR8ko mice led to total protection from SS disease, demonstrating that the SS phenotype in TLR8-deficient mice is TLR7-dependent. Therefore, in the non-autoimmune prone C57BL/6 background increased TLR7 signaling by DCs is central for SS disease manifestations in both genders.

## Implication of TLR7 signaling in patients with primary SS

Recent studies sought to obtain insights into the potential participation of TLR activation in pSS, and most of them focused on the evaluation of TLR7, TLR8 and TLR9 expression in human peripheral blood mononuclear cells (PBMCs) and SGs. Evaluation of PBMCs from 20 patients with pSS showed that TLR7 protein levels were significantly higher in pSS patients, whereas the mRNA levels of TLR8 and TLR9 were higher and lower, respectively, than in healthy controls (92). Additionally, a study with 20-37 pSS patients revealed increased TLR7 and TLR9, and normal TLR8, mRNA levels in the PMBCs of pSS patients (93). In the same study, TLR7 and TLR9 expression was detected in the epithelial islands, lymphocytes and ductal epithelial cells of the parotid salivary glands of the pSS patients, while in healthy controls their expression was limited to ductal epithelial cells (93). A study with 115 pSS patients demonstrated upregulation of TLR7, but not TLR9, in circulating pDCs and monocytes in IFN-positive patients, as well as, TLR7 expression within the lymphocytic foci in the SGs (94). Additionally, the TLR7 downstream signaling molecules MyD88, IRF7 and RSAD2 (a marker of DC maturation) were also upregulated, suggesting an increased TLR7 signaling. In accordance to these findings, we reported that in a cohort of 40 pSS patients TLR7 mRNA levels were significantly overexpressed in the minor SGs compared to healthy controls, and importantly TLR7 overexpression was positively correlated to TNF, LTα, CXCL13 and CXCR5 expression (90). Additionally, in another independent study immunohistochemistry analysis of labial SGs (a subset of minor SG) from 11 pSS patients revealed high TLR7 expression in infiltrating mononuclear cells and ducts and weak TLR9 and TLR8 expression in ducts and infiltrating mononuclear cells, respectively (95). Notably, TLR7 and its downstream molecules MyD88, TRAF6 and IRF-7 were highly expressed mainly in pDCs of labial SGs in pSS patients (95). Overall, these studies identified mainly an increased TLR7 expression and signaling in the PBMCs and salivary glands of SS patients, while the implication of TLR8 and TLR9 seem to be less evident.

B cells have an important role in SS and detection of autoantibodies against SSA/Ro or SSB/La is one of the diagnostic criteria. Thus, some studies have focused on the implication of TLRs on B cell function in the context of pSS. Assessment of TLR7 and TLR9 expression in B cell subsets of 25 patients with pSS by FACS analysis did not detect significant disparities in TLR7 and TLR9 expression between the two cohorts (96). This is not surprising since the protein levels of TLR7 and TLR9 are quite low in human, as well as in mouse, B cells which makes their detection rather difficult (97). On the other hand, transcriptomic analysis of CD19+ B cells from 12 anti-SSA antibody-positive untreated patients with pSS revealed increased expression of TLR7, as well as, type I and II IFN-induced genes, chemokines and chemokine receptors compared to healthy donors (98). Analysis of the responsiveness of B cells isolated from peripheral blood of 21 pSS patients to TLR7 and TLR9 ligands revealed increased levels of secreted cytokines in pSS patients. In particular, TLR7 stimulation led to increased IFNα production, while TLR9 stimulation induced elevated IL-8, IL-15 and MCP-1 levels in pSS patients (99). Moreover, pSS patients had more naïve B cells, less pre-switched memory B cells and fewer IL-10 positive pre-switched memory B cells compared to healthy controls, both in untreated and TLR7 stimulated cells (99). Importantly, murine B cells can negatively regulate immunity through provision of IL-10 during the course of autoimmune and infectious diseases, and thus human B cells might also perform similar inhibitory functions through the provision of IL-10 (100). Additionally, a study with 25 pSS patients reported increased signaling potential in peripheral B cells of pSS patients in response to combined TLR7 and TLR9 stimulation through STAT3 and NF-κB phosphorylation that correlated with a type I IFN signature (101). pSS-associated thrombocytopenia is present in 7-12% of pSS patients and seriously affects quality of life and life expectancy (102, 103). A study with 3 patients with pSS-associated thrombocytopenia, 3 pSS patients with normal platelets and 3 healthy controls, revealed increased TLR7 and IL-8 expression in B cells of pSS-associated thrombocytopenia compared to pSS patients without thrombocytopenia or healthy controls (104). Notably, in the same study activation of the TLR7 pathway in NOD mice led to thrombocytopenia and increased IL-1β and IL-8 levels both in the serum and submandibular glands compared to control groups (104). These data demonstrate that elevated TLR7 expression and type I IFN signaling by B cells is characteristic in pSS patients, and coincide with the recent discoveries that type I IFN production by B cells, and not only by pDCs, is important in the pathogenesis of SLE (105).

The implication of TLR7/8 signaling in the pSS development has been also highlighted through the detection of endogenous retroelements (106). Endogenous retroelements (viruses present in the human genome) are important source of nucleic acids, can be detected by TLRs and play essential role to the proper function of the immune system, as well as, in the pathogenesis of immune disorders, including autoimmunity (107, 108). In this context, it was shown that in minor salivary gland tissues derived from 31 pSS patients there was an upregulation of the long interspersed nuclear element 1 (LINE-1), an autonomous family of retroelements that remain active in mammalian genomes compared to controls (106). This increased LINE-1 expression was in strong correlation with type I IFN transcripts in pSS patients. Moreover, *in vitro* experiments revealed that expression of type I IFN regulated genes in pDCs stimulated with LINE-1 retroelements was abrogated by a TLR7/8 inhibitor (106). Thus, the detection of endogenous retroelements by TLR7/8 may contribute to initiation or amplification of pSS. Collectively, increased TLR7 expression and/or signaling by PBMCs, pDCs, B cells and/or monocytes has been detected in pSS patients in various independent studies from different labs and cohorts supporting the idea that TLR7 signaling is critical in SS development.

All the above human studies have been performed exclusively or mainly in female pSS patients, which mirrors the fact that SS occurs predominately in women, with a female to male ratio 9-20:1 (5). The female bias in SS and other autoimmune disease, including SLE, is incompletely understood and most likely multifactorial. Although, sex hormones play an important role, recent work suggests that X chromosome gene dosage is also an important driver of sex bias in autoimmunity (109, 110). In this regard, in both humans and mice the TLR7 and TLR8 genes are located on the short arm of the X chromosome and in a region that escapes X chromosome inactivation (111). In fact, a study with 1,033 female pSS patients revealed a 2.9 higher prevalence of trisomy X in the pSS patients (47, XXX) than in female controls (46, XX) (112). In similar fashion, evaluation of the frequency of Klinefelter’s syndrome (47, XXY) in 136 male pSS patients found increased incidence of Klinefelter’s syndrome in pSS patients compared to healthy men (113). Moreover, rare X chromosome abnormalities with triplication of the distal p arm of the X chromosome were found in both patients with pSS or secondary SS (114). Noteworthy, recent studies revealed that TLR7 escapes from X chromosome inactivation in pDCs, B cells and monocytes of healthy women, as well as of Klinefelter’s males and hence it is possibly implicated in the enhanced susceptibility of women and Klinefelter’s syndrome men to systemic autoimmunity (115, 116). Furthermore, IFNα production by pDCs following TLR7 stimulation shows a strong sex bias in humans and mice (117–119). In line to these data, we reported previously significant higher expression of TLR7 mRNA levels and more profound SG inflammation in the salivary glands of female versus male TLR8ko mice that spontaneously develop secondary SS due to increased TLR7 expression by DCs (90). Thus, higher TLR7 expression and signaling in women than in men can predispose women to SS, and in part explain the sex bias in autoimmunity.

Collectively the data currently suggest that TLR7 is implicated in the progression and probably development of pSS in humans. However, given the fact that most of the studies have been performed in small number of patients from urban settlements, where usually the individuals maintain high hygiene standards and are exposed to low pathogenic burdens, further studies focusing in more diverse populations are needed to increase the validity of the findings.

## A hypothetical but possible connection between SARS-CoV-2 and SS through TLR7

In general, viral or bacterial infections are considered as an important environmental factor that accelerate the incidence and/or disease activity of autoimmune diseases. In the case of SS, Epstein-Barr virus, cytomegalovirus, hepatitis C virus, coxsackie virus, human T-lymphotropic virus, retroviruses and enteroviruses are considered as critical environmental triggers (120). Lately, the severe acute respiratory syndrome coronavirus 2 (SARS-CoV-2), which caused the coronavirus-associated acute respiratory disease (COVID-19) pandemic, represents a new source of concern as a causative agent for triggering and managing autoimmune diseases, including SS (121, 122). SARS-CoV-2 belongs to the coronavirus family, which are large, enveloped viruses with spherical morphology, prominent glycoprotein spikes and a ssRNA genome (123). SARS-CoV-2 binds to various cell types *via* the angiotensin-converting enzyme 2 (ACE-2) molecules expressed on the cell surfaces. Upon binding, viral RNA enters the cytosol of the infected cell and stimulate the production of pro-inflammatory cytokines including IL-6, TNF, IL-18, IL-1 and IFNs (IFNα, IFNγ and IFNλ) causing substantial inflammatory reactions (124). In susceptible patients, the uncontrolled release of inflammatory cytokines, called cytokine storm, can lead to severe acute respiratory distress syndrome (124). AEC2 is highly expressed on the mucosa of oral cavity and the salivary glands (125, 126), and as a consequence SARS-CoV-2 is able to infect and replicate in salivary glands (127). Importantly, a study in Brazil, which has been deeply affected by the COVID-19 pandemic, reported that the number of newly diagnosed SS cases was increased by 50,7% during the 2020 COVID-19 period compared to the 2017-2019 period (128). Thus, SARS-CoV-2 probably acts as a new critical environmental trigger for SS development. SARS-CoV-2 can disturb self-tolerance and trigger autoimmune responses through cross-reactivity with host cells (122), and thus, we could hypothesize that TLR7 signaling is possibly implicated in this process. Indeed, TLR7 detects SARS-CoV-2 ssRNA and is crucial for the production of antiviral interferons, while the implication of TLR3 and TLR8 seems to be less profound (129, 130). Moreover, TLR7 signaling plays a key protective role during SARS-CoV-2 infection, since loss-of-function variants in the X-linked TLR7 leads to life threatening COVID-19 disease in young males (129, 131, 132). At the same time, robust and uncontrolled TLR7 signaling can contribute to a cytokine storm and sever COVID-19 as well as to autoimmunity. Indeed, autoantibodies occur in a number of autoimmune diseases and patients with COVID-19 exhibit increased autoantibody reactivities as compared to uninfected individuals (122, 133). Notably, in a case report the presence of high anti-Ro/SSA antibody titers in two patients with aggravated COVID-19 pneumonia, but without previous history of autoimmune disease, advocate to the hypothesis that the patients developed aggravated COVID-19 pneumonia due to an autoimmune response (134). It is possible that SARS-CoV2 contributes to the immunopathology of SS, not only through TLR7 signaling, but also through other pattern recognition receptor signaling pathways, such as the activation of the cGAS-STINK pathway which can be activated by DNA that emerges from a collateral host response to viral tissue damage (135). In fact, SARS-CoV2 infection can drive thought the cGAS-STING pathway a type I IFN and/or NF-κB immunopathology in COVID-19 (136, 137). Overall, based on the above presented data we can hypothesize that SARS-CoV-2 could be a causative agent for SS, probably through the activation of the TLR7 signaling pathway, since the virus can be detected by TLR7 and TLR7 signaling is essential for the antiviral immune response of the host in COVID-19. This novel hypothesis requires further validation through collection of more data that will arise in the future.

## Therapeutic approaches targeting nucleic acid-sensing TLRs

The ability to specifically target TLRs for the prevention and treatment of autoimmune and inflammatory diseases, as well as some cancers, viral and bacterial infections is of great interest. Various new compounds that target TLRs are currently undergoing preclinical and clinical evaluation (138, 139). Here, we will focus on current advances regarding nucleic acid-sensing TLR inhibition for the treatment of autoimmune diseases (**Table 1**).

**Table 1:** Compounds in development that target human nucleic acid-sensing TLRs for autoimmunity.

Compound	Drug class	Target	Indications	Company	Status/Trial ID	Reference
CQ/HCG	Small molecule/Inhibitor	TLR7, TLR8 and TLR9	SLE, RA	Generic	Clinical use	(140)
			SS		Phase III NCT016001028 NCT00632866	(141)
M5049 (Enpatoran)	Small molecule/Inhibitor	TLR7 and TLR8	SLE	Merck	Animal study	(142)
			SLE		Phase I NCT03676322	(143)
			SLE, CLE		Phase I NCT04647708	
			SLE, CLE, SCLE, DLE		Phase II NCT05162586	
			COVID-19 pneumonia		Phase II NCT04448756	
IRS-954	Oligonucleotide	TLR7 and TLR9	SLE	–	Animal study	(144, 145)
IMO-3100	IMO	TLR7 and TLR9	psoriasis	Idera Pharmaceuticals	Phase II NCT01622348	
IMO-8400	IMO	TLR7, TLR8 and TLR9	SLE, psoriasis	Idera Pharmaceuticals	Animal study	(146–148)
			psoriasis		Phase IIa NCT01899729	(149)
			cancer		Preclinical, Animal study	(150)
			B cell lymphoma		Phase I/II NCT02363439 NCT02252146	
BMS-986256 (Afimetoran)	Small molecule	TLR7 and TLR8	SLE	Bristol-Myers Squibb	Phase I/II NCT04895696	(151, 152)
DS-7011a	Antibody	TLR7	SLE	Daiichi Sankyo	Phase I NCT05203692	

CLE, cutaneous lupus erythematosus; CQ, chloroquine; DLE, discoid lupus erythematosus; HCQ, hydroxychloroquine; IMO, Immune modulatory oligonucleotide; SCLE, subacute cutaneous lupus erythematosus; SLE, systemic lupus erythematosus.

Small molecule inhibitors are synthetic or naturally derived chemical agents with amphipathic property that allow them to cross cell membranes easily, and once inside the cell can act on proteins or compartments and affect TLR signaling pathways. Antimalaria drugs, such as hydroxychloroquine (HCQ) and chloroquine (CQ), are small molecule inhibitors that are known to act as a TLR9, and to a lesser extend as TLR7 and TLR8, antagonist, and are currently used as monotherapy or in conjunction with other therapies for the treatment of SLE, rheumatoid arthritis and SS (140). However, two independent Phase III clinical trials with daily administration of HCQ for 12 weeks (NCT016001028) or 24 weeks (NCT00632866) showed no clinical benefits for dry eyes and systemic inflammation in pSS patients (141, 153). At the present time, a clinical trial on whether blood levels of HCQ in patients with primary SS could influence the therapeutic response is ongoing (NCT04546542). A new promising agent that inhibits both TLR7 and TLR8 signaling by blocking synthetic and natural endogenous RNA ligands is M5049 (Enpatoran). This compound prevents SLE progression in SLE-prone mice and reduces serum levels of autoantibodies (142). In a Phase I clinical trial (NCT03676322), M5049 was well tolerated by healthy participants (143). Currently, M5049 is being evaluated in a Phase I (NCT04647708) clinical trial for SLE, and cutaneous lupus erythematosus CLE, and in a Phase II (NCT05162586) clinical trial in SLE, CLE, subacute cutaneous lupus erythematosus (SCLE) and/or discoid lupus erythematosus (DLE) participants. Additionally, a Phase II clinical trial revealed promising results regarding the efficacy of M5049 on severe inflammatory symptoms in patients with COVID-19 pneumonia (NCT04448756).

Oligonucleotides with certain sequences are predicted to function as antagonists of nucleic acid-sensing TLRs by interfering with the binding of the nucleic acid ligands to the TLRs. One such oligonucleotide-based TLR antagonist, also termed immunoregulatory DNA sequence (IRS), is IRS-954. This oligonucleotide with a dual-function on targeting both TLR7 and TLR9 is able to reduce autoantibodies and improve disease symptoms in lupus-prone mice (144, 145) as well as to reverse TLR7/TLR9-mediated glucocorticoid resistance of SLE in mice, suggesting a potential therapeutic opportunity as corticosteroid drug (154). Despite the promising pre-clinical therapeutic efficacy of IRS-954, no clinical development has been reported so far.

Other oligonucleotide-based TLR antagonists, also named immune modulatory oligonucleotides (IMO), containing a synthetic immunostimulatory dinucleotide and a novel DNA structure have been demonstrated to have therapeutic potential on autoimmune diseases. One such molecule, IMO-3100 is a TLR7 and TLR9 dual antagonist that inhibits inflammation in an IL-23-induced skin inflammation model in mice (155), while in a Phase II clinical trial (NCT01622348) it demonstrated positive results in reducing disease severity in patients with moderate to severe plaque psoriasis. IMO-8400 is a second-generation oligonucleotide antagonist that inhibits TLR7, TLR8 and TLR9 as well as disease progression of SLE and psoriasis in animal models (146–148). In a Phase IIa clinical trial in patients with moderate to severe psoriasis, short-term IMO-8400 treatment led to reduced psoriasis severity (149). Furthermore, in the setting of cancer, IMO-8400 inhibited the oncogenic mutant L265P in the MyD88 gene, which leads to increased TLR signaling, thereby interrupting the proliferation of cell populations (150). In Phase I/II clinical trials, the compound was tested as treatment on patients who harbor the specific oncogenic MyD88 L265P mutation, and develop defined forms of B-cell lymphoma, such as Waldenstrom’s macroglobulinemia (NCT02363439) or diffuse large B-cell lymphoma (NCT02252146), and the results seem promising. Another type of oligonucleotide antagonists for TLRs are the prototypic inhibitory oligonucleotide (INH-ODN) 2088 as well as a set of improved guanine-modified INH-ODNs (156). These improved INH-ODNs can inhibit TLR7- and TLR9-induced immune responses both in mouse and human cells, but their ability to block TLR8 signaling has not been addressed (156, 157).

Research for small molecules that could inhibit nucleic acid-sensing TLR activity has also been promising. As such, 7f is a small molecule antagonist of TLR7, TLR8 and TLR9, orally bioavailable that promotes reduction of proteinuria, anti-dsDNA autoantibody titers and serum IL-10 in MRL/lpr lupus prone mice (158). Moreover, BMS-986256 (Afimetoran) is another small molecule that blocks TLR7 and TLR8, can be delivered orally and was able to prevent lupus symptoms in animals treated before the onset of disease as well as reverse organ damage when given after symptoms had already started (151). Two Phase I clinical trials confirmed that BMS-986256 is well tolerated at a range of doses in healthy volunteers (151, 152), and currently it is in Phase II clinical development (NCT04895696) for the treatment of SLE.

An additional approach for dampening TLR function is the development of antibodies that target specific TLRs. Indeed, administration of an antibody against TLR7 that could block TLR7 signaling protected lupus-prone NZBWF1 mice from nephritis and inhibited autoantibody production (159). Nevertheless, anti-TLR9 antibody administration did not ameliorate lupus disease, suggesting that TLR9 is dispensable for disease progression in NZBWF1 mice (159). Currently, an antibody (DS-7011a) that blocks human TLR7 is in a Phase I clinical trial (NCT05203692).

All the approaches mentioned above are based on blocking the binding of TLR ligands to the receptor, however, another strategy to achieve TLR inhibition is by targeting the downstream TLR signaling pathways to stop signal transduction and mainly type I IFN production, since it plays a significant role in the pathogenesis of SS and is correlated with SS clinical features (15, 160). Based on this, and because type I IFNs signal through the Janus kinase/signal transducer and activator of transcription (JAK/STAT) pathway to induce IFN-stimulated gene expression, therapeutic inhibitors of the JAK/STAT pathway have appeared as potentially new drugs in SS and other type I IFN-mediated autoimmune diseases, and are currently in early-phase clinical trials (161, 162). However, the type I IFN pathway is activated not only by nucleic acid-sensing TLRs, but also by other nucleic acid sensors, including GAS, RIG-I and MDA-5. Thus, inhibitors that block TLR activity at the level of the receptor probably have lower anti-inflammatory potential, but also fewer off-target effects. On the contrary, inhibitors that target downstream proteins implicated in several pathways can cause greater toxicity that will result in the discontinuation of promising drug candidates.

Currently, while various clinical trials test the safety, efficacy and stability of TLR inhibitors (at the receptor or signaling level), of which some show positive results, one major concern is whether these new therapies could impair host’s immune defense. Indeed, microbes often activate multiple TLRs as well as the type I IFN signaling pathway. Thus, dampening these innate arms of defense can leave the host unprotected in the likely case of viral or bacterial infection. Therefore, it is tempting to postulate that targeting one TLR, instead of many TLRs or their downstream signaling pathways, might be a more precise approach and with less dramatic side-effects, like an immunocompromised host against microbes.

## Concluding remarks

In the past two decades the intense research in mammalian TLRs regarding their characterization, ligand recognition, signaling and biological function in host defense and disease development has progressed enormously. Nucleic acid-sensing TLRs, through the detection of microbial and/or endogenous nucleic acids are critical in shaping innate and adaptive immunity, as well as in driving autoimmune and inflammatory diseases when they are dysregulated. Despite the variance between humans and mice, including the expression and distribution of TLR7, TLR8 and TLR9 in different cell types, there are numerous accumulating evidence that TLR7 signaling plays a key role in SS pathogenesis. Notably, TLR8 and TLR9 act not only as signaling receptors, but also as important regulators of TLR7 expression and signaling, whereas in the mice TLR8 restrains TLR7 in DCs, while TLR9 constrains TLR7 in B cells (50). The importance of this regulation is reflected by the fact that deletion of TLR8 or/and TLR9 in mice or even diminution of TLR8 expression in humans, leads to increased TLR7 signaling and subsequent autoimmunity (50, 54, 57). The current progress on the development of various compounds that target specific nucleic acid-sensing TLRs, which are currently in preclinical and clinical evaluation (**Table 1**), will probably open the way to new approaches on treating TLR-dependent autoimmune diseases, including SS. Based on the importance of TLR8 and TLR9 in restraining TLR7, special attention has to be given, when therapeutic approaches are targeting the blocking of one versus another nucleic acid-sensing TLR in order to avoid unwanted consequences of disbalanced and unwanted TLR7 signaling, which can be detrimental.

## Author contributions

The author confirms being the sole contributor of this work and has approved it for publication.

## Funding

This work received funding to LA from the Agence Nationale de la Recherche (ANR-18-CE15-0022). We also acknowledge institutional support from CNRS, INSERM and Aix-Marseille University.

## Conflict of interest

The author declares that the research was conducted in the absence of any commercial or financial relationships that could be construed as a potential conflict of interest.

## Publisher’s note

All claims expressed in this article are solely those of the authors and do not necessarily represent those of their affiliated organizations, or those of the publisher, the editors and the reviewers. Any product that may be evaluated in this article, or claim that may be made by its manufacturer, is not guaranteed or endorsed by the publisher.
